# Tryptophan Suppresses *FTH1* ‐Driven Ferritinophagy, a Key Correlate of Prognosis in Hepatocellular Carcinoma

**DOI:** 10.1111/cpr.70074

**Published:** 2025-06-12

**Authors:** Xinxiang Cheng, Xin Ge, Chi Zhang, Xingye Yang, Zhengxin Yu, Min Zhang, Wen Cao, Qingtao Ni, Yang Liu, Songbing He, Yin Yuan

**Affiliations:** ^1^ Department of General Surgery Wuxi No. 2 Chinese Medcine Hospital Wuxi China; ^2^ Department of Emergency Medicine Wuxi No. 2 Chinese Medcine Hospital Wuxi China; ^3^ Department of Hepatobiliary Surgery The Affiliated Taizhou People's Hospital of Nanjing Medical University, Taizhou School of Clinical Medicine, Nanjing Medical University Taizhou China; ^4^ Department of Liver Disease The Affiliated Taizhou People's Hospital of Nanjing Medical University, Taizhou School of Clinical Medicine, Nanjing Medical University Taizhou China; ^5^ Department of Oncology The Affiliated Taizhou People's Hospital of Nanjing Medical University, Taizhou School of Clinical Medicine, Nanjing Medical University Taizhou China; ^6^ Department of Clinical Research Center The Affiliated Taizhou People's Hospital of Nanjing Medical University, Taizhou School of Clinical Medicine, Nanjing Medical University Nanjing China; ^7^ Department of General Surgery The First Affiliated Hospital of Anhui Medical University Hefei China; ^8^ Department of General Surgery The First Affiliated Hospital of Soochow University, Cancer Institute, Suzhou Medical College, Soochow University, Suzhou Biomedical Industry Innovation Center & National Center of Technology Innovation for Biopharmaceuticals Suzhou China

**Keywords:** autophagy, ferritinophagy, *FTH1*, hepatocellular carcinoma, reactive oxygen species, tryptophan

## Abstract

Hepatocellular carcinoma (HCC) remains a lethal malignancy with limited therapeutic options. Ferritinophagy, an autophagy‐dependent process regulating iron metabolism, has emerged as a key contributor to ferroptosis and tumour progression. This study hypothesised that the ferritinophagy‐related gene *FTH1* drives HCC pathogenesis by modulating tryptophan metabolism and reactive oxygen species (ROS)‐dependent ferroptosis. To test this, we first analysed TCGA data to identify prognostic ferritinophagy genes, revealing *FTH1* as a critical risk factor. Functional experiments using *FTH1*‐knockdown/−overexpressing HCC cell lines and xenograft models demonstrated that *FTH1* enhances proliferation, migration, and tumour growth by upregulating CYP1A1/CYP1A2 in the tryptophan pathway, thereby increasing the synthesis of 6‐hydroxymelatonin (6‐HMT). Mechanistically, 6‐HMT suppressed ROS and ferroptosis by inhibiting cytochrome P450 oxidoreductase (POR). Concurrently, intracellular tryptophan levels were found to inhibit NCOA4‐mediated selective autophagy of FTH1, stabilising FTH1 levels and promoting tumour survival. Collectively, our findings establish *FTH1* as a central regulator of ferritinophagy in HCC and reveal its dual role in linking tryptophan metabolism to redox homeostasis. This result provides a hint of how *FTH1* influences HCC pathogenesis and positions the tryptophan metabolism pathway as a promising therapeutic target.

## Graphical abstract

Intracellular tryptophan levels modulate selective autophagy of FTH1 via the NCOA4 pathway. This autophagic process then inhibits 6‐HMT synthesis, which then results in an increase in reactive oxygen species and ferroptosis.

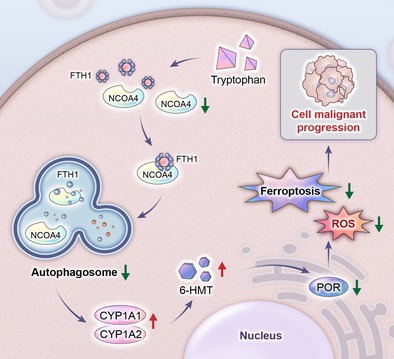

## 1 Introduction

Hepatocellular carcinoma (HCC) is a common cancer in adults with a 17.65% incidence and a 15.07% mortality rates [1]. The available treatments include surgical intervention, chemotherapy, radiotherapy, and interventional therapy. However, HCC has a poor prognosis and a notably low 5‐year survival rate, with the majority of patients diagnosed at an advanced stage. A high hepatic arterial coefficient and lymphatic density significantly predicted poorer overall and recurrence‐free survivals in HCC patients [2, 3]. For individuals with advanced HCC, targeted therapy and immunotherapy are now available, but the prognosis remains unfavourable [4]. The pathogenic mechanisms and pathophysiological processes in HCC present major challenges. To advance therapeutic strategies, investigations into novel molecular mechanisms underlying HCC progression are required.

Ferroptosis is an iron‐dependent mode of cell death characterised by lipid peroxidation and the consequent production of reactive oxygen species (ROS) [5]. Ferroptosis is involved in the pathogenesis of various diseases, including cancers, ischaemia–reperfusion injury, degenerative diseases, and blood disorders [6, 7]. Ferroptosis onset involves a novel form of autophagy called ferritinophagy, characterised by ferritin degradation. Ferritin is a 24‐subunit globular protein comprising a heavy chain (FTH1) and a light chain (FTL) capable of storing up to 4500 iron atoms [8]. Ferritin synthesis is upregulated in response to elevated intracellular iron levels [9]. Conversely, ferritin is degraded at low iron concentrations, resulting in the release of sequestered iron [10]. This process is facilitated by nuclear receptor coactivator 4 (NCOA4), which binds ferritin and transports it to autophagosomes [9] for fusion with lysosomes [10].

Ferritinophagy contributes to oxidative damage and ferroptosis via the Fenton reaction [11]. Generally, autophagy products contribute to cellular remodelling and homeostasis by providing energy and essential nutrients for cell development [12]. Hence, impairment of this process is likely to play a crucial role in disease. Investigation of the regulatory mechanisms and functional roles of ferritinophagy can contribute to a deeper understanding of the pathogenesis in cancers such as HCC, in addition to identifying novel therapeutic targets.

Recent evidence has highlighted the interplay between ferritinophagy and amino acid pathways in cancer progression [13]. The essential amino acid tryptophan is metabolised into bioactive compounds that regulate immune responses [14], redox balance [15], and tumour proliferation [16]. Notably, tryptophan‐derived metabolites such as 6‐hydroxymelatonin (6‐HMT) exhibit antioxidant properties, thereby potentially counteracting ferroptosis. However, whether ferritinophagy intersects with tryptophan metabolism to influence HCC progression remains unexplored. We hypothesize that *FTH1*, a key ferritinophagy regulator, drives HCC pathogenesis by reprogramming tryptophan metabolism to suppress ROS and ferroptosis, thereby creating a pro‐tumorigenic environment.

## 2 Materials and Methods

### 2.1 Bioinformatics Analysis

Transcriptomic data of 374 HCC cases and 50 normal controls from The Cancer Genome Atlas (TCGA) were downloaded along with clinical and prognosis‐related information. Cases were excluded if the survival status, TNM stage, or overall survival were unavailable. Ferritinophagy‐related genes were identified via a search for “ferritinophagy” in GeneCards (http://www.genecards.org/). Genes absent during probe conversion were excluded from the subsequent analysis. Differentially expressed ferritinophagy‐related genes were subjected to one‐way Cox regression analysis in R, with an adjusted *p*‐value < 0.05. Genes meeting this criterion were visualised in a forest plot using the R package “forestplot.” A multifactorial analysis was conducted using the R package “survival” to explore the correlation between ferritinophagy‐related genes and patient prognosis. Gene–immune cell interactions were further analysed using the R package “ggpubr.”

### 2.2 Patients and Samples

The study randomly obtained 120 tumour sections from patients who underwent radical surgery for HCC at the First Affiliated Hospital of Soochow University between January 2011 and December 2013. The enrollment criteria were as follows: pathohistological confirmation of HCC post‐surgery, no antitumour treatment before surgery, and the presence of comprehensive follow‐up data. All participants provided informed consent and the study was approved by the hospital's ethics committee.

### 2.3 RNA Extraction and Quantitative Real‐Time PCR

Total RNA was extracted using RNA extraction solution and treated with trichloromethane for 3 min at room temperature. RNA concentrations were determined spectrophotometrically using a NanoDrop 2000 device.

All PCRs were conducted using 2× SYBR Green qPCR Master Mix (Roche, USA) and a PE 9700 thermocycler (PE Applied Biosystems, Foster City, CA, USA). The thermocycling schedule was as follows: 95°C for 5 min, followed by 40 cycles of 95°C for 15 s, 60°C for 20 s, and 72°C for 40 s. The primer sequences were synthesised by Shanghai Sangon Biological Engineering (Shanghai, China); see Supplementary Table 1.

### 2.4 Immunohistochemistry

Fixed tumour sections (4 μm thick) were stained with anti‐human polyclonal Ki67 and FTH1 antibodies, followed by incubation with horseradish peroxidase‐conjugated secondary antibodies. The sections were incubated with developing solution and counterstained with haematoxylin. The stain was stabilised using an acid alcohol, followed by dehydration.

### 2.5 Immunofluorescence

Samples were incubated overnight at 4°C with the appropriate primary antibody, washed with phosphate‐buffered saline (PBS), and incubated with specific fluorescence‐conjugated secondary IgG for 50 min at room temperature. Sections were counterstained with DAPI and mounted for analysis. The immunofluorescence signals were imaged with a fluorescence microscope.

### 2.6 Cell Lines and Transfections

Hepatocellular carcinoma cell lines (Huh‐7, HepG2, and Hep3B) and a normal human liver cell line (LO2) were obtained from the American Type Culture Collection. Constructs for the empty vector, overexpression, and lentiviral packaging plasmids were generated. The transfection efficiency was assessed 48 h post‐transfection by fluorescence microscopy, and overexpression was quantified by quantitative reverse transcription PCR (qRT‐PCR).

### 2.7 Transwell Assay

Cell migration and invasion were evaluated by Transwell assay. Cells were fixed with 4% paraformaldehyde, observed with a 200× microscope, and images of five randomly selected fields of view were recorded.

### 2.8 Tumour Xenograft Model

Wild‐type (WT), *FTH1* knockdown (sh_*FTH1*), and *FTH1* overexpressing (ov_*FTH1*) HepG2 cells (3 × 10^6^ cells/group) were injected subcutaneously into 4‐week‐old female BALB/c mice. Tumour dimensions in the axillary region were monitored and recorded beginning on day 7 post‐injection. After 21 days, the mice were euthanized and the implanted tumours were surgically excised and then preserved for immunohistochemical analyses.

### 2.9 Protein Extraction and Western Blotting

Protein extraction was performed using RIPA lysis buffer. The protein concentrations were quantified by bicinchoninic acid (BCA) protein assays (Procell Life Science & Technology, Wuhan, China). The proteins were separated by SDS‐PAGE and transferred onto polyvinylidene fluoride (PVDF) nanofibrous membranes. Immunoreactive proteins were detected with enhanced chemiluminescence (ECL, ProCell Life Science & Technology, Wuhan, China).

### 2.10 RNA‐Seq and Transcriptomic Profiling

RNA sequencing libraries were prepared using a KAPA Stranded mRNA‐Seq Kit and quantified using real‐time quantitative PCR. Sequencing was performed on an Illumina HiSeq platform. Splice sequences were removed from the raw downstream data with Cutadapt, and low‐quality sequences were eliminated using Trimmomatic. Next, FastQC was used to calculate the data quantity, as well as the Q20 and Q30 ratios. The cleaned data were aligned with the reference genome in HISAT. Aligned reads were assembled into transcripts and analysed with StringTie.

### 2.11 Metabolome Analysis

A non‐targeted metabolomics approach was adopted. Specifically, 50 mg of WT and sh_*FTH1* HepG2 cell samples were weighed and mixed with 400 μL of extraction solution (methanol: water = 4:1). To facilitate extraction, the mixture was vortexed for 30 s and subjected to low‐temperature sonication for 30 min (5°C, 40 kHz). The extracted samples were allowed to stand for 30 min before centrifugation (13,000 × *g* and 4°C for 15 min). Differential metabolites in the collected supernatant were analysed by liquid chromatography–mass spectrometry.

### 2.12 Coimmunoprecipitation (CO‐IP) Assay

A CO‐IP kit (Thermo Fisher Scientific) was used. Cell lysate supernatant was incubated overnight with specific antibodies and then with A/G beads. After washing and centrifugation, the eluates were subjected to SDS‐PAGE and western blotting.

### 2.13 5‐Ethynyl‐2′‐Deoxyuridine (EdU) Staining

A Cell‐Light EdU DNA Cell Proliferation Kit (RiboBio) was used for EdU assays. Cells were seeded into 96‐well plates and cultured for 24 h. Following a 2 h incubation in 50 μM EdU, the cells were treated with 50 μL of fixative and stained with Apollo Dye Solution. Nuclei were counterstained with DAPI. Three random fields of view per well were imaged with a 200× microscope, avoiding overlapping or edge regions to ensure unbiased sampling. Quantification of EdU‐positive cells was performed using ImageJ software (NIH) by thresholding fluorescence intensity (EdU signal > 2× background) and normalising to the total DAPI‐stained nuclei. The data are expressed as the percentage of EdU‐positive cells.

### 2.14 Measurement of ROS Levels

The oxidation‐sensitive fluorescent probe DCFH‐DA and a ROS Assay Kit (Jianglaibio, Shanghai, China) were used to assess ROS levels.

### 2.15 Tryptophan and 6‐HMT Measurements

Cells (*n* = 100,000) were seeded in 12‐well plates and incubated in DMEM supplemented with 10% FBS for 10 h. The cells were lysed by repeated freeze–thaw cycles. Next, the intracellular tryptophan and 6‐HMT concentrations were determined using the respective assay kits.

### 2.16 Measurement of Autophagy Levels

To assay autophagic vesicle levels, Huh‐7 cells were transfected with adenoviruses at a multiplicity of infection (MOI) of 100. After 24 h of culture, fluorescent signals in the Huh7 cells were imaged using confocal microscopy.

### 2.17 Electron Microscopy

A 1 × 1 × 1 mm^3^ piece of tissue was fixed in 2.5% glutaraldehyde. After dehydration using an alcohol gradient, samples were embedded in Epon 812 resin. Semithin sections were prepared using an EM UC7 ultramicrotome (Leica, Wetzlar, Germany), stained with 2% uranium acetate and lead citrate, and examined using a transmission electron microscope (Hitachi HT7800).

### 2.18 Statistical Analysis

The data are presented as means ± standard deviation. The standard error of the mean (SEM) was calculated from the mean of at least three independent samples per condition. The data were analysed using an unpaired Student's *t*‐test or one‐way analysis of variance (ANOVA) with Tukey's multiple comparison test. Survival analysis was performed using the Kaplan–Meier method. All analyses were performed in GraphPad Prism 8.0 (GraphPad Software). Significance was set at *p* < 0.05.

## 3 Results

### 3.1 *FTH1* Is a Key Ferritinophagy Gene in HCC

**FIGURE 1:**
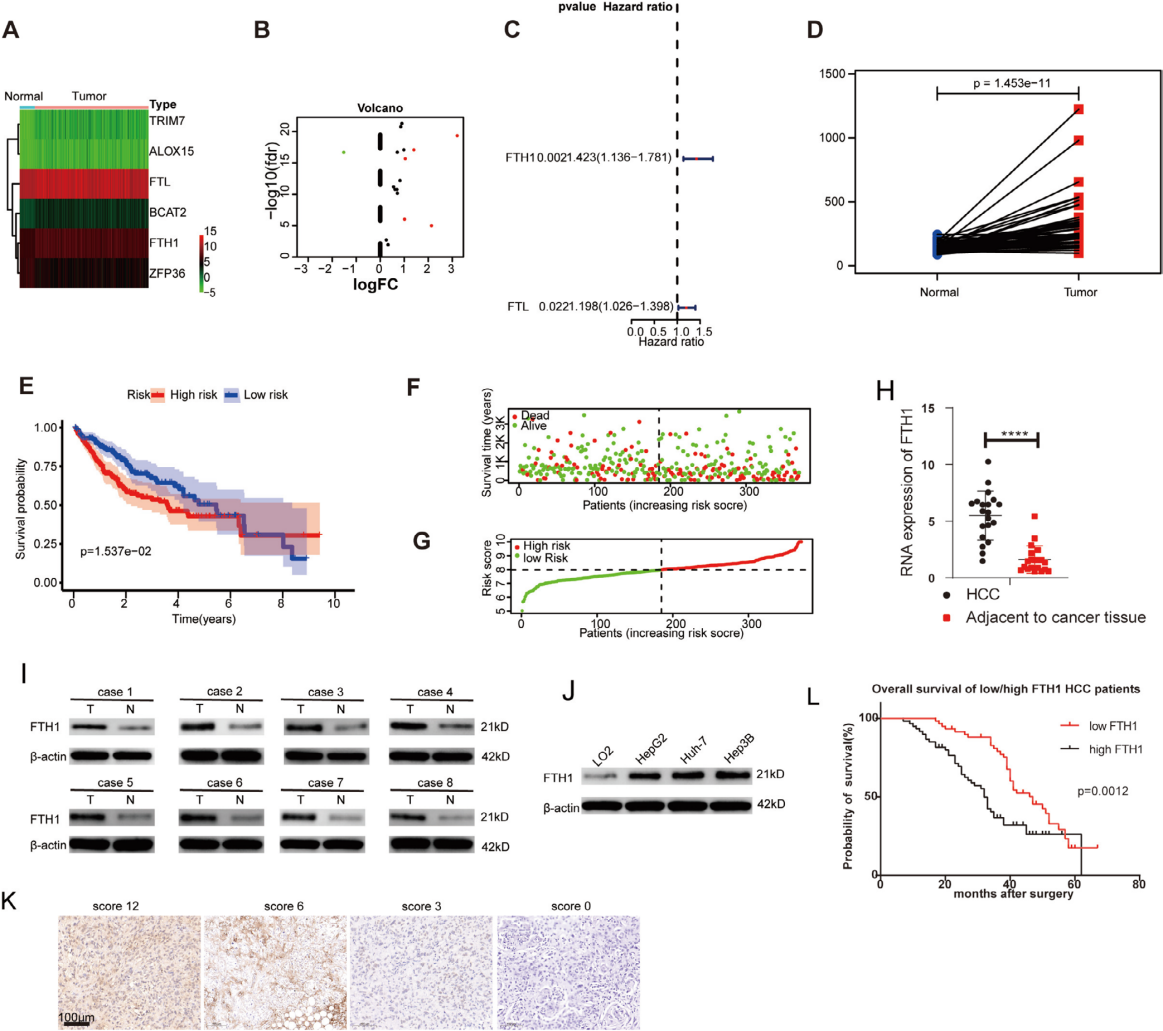
*FTH1* is a key ferritinophagy gene in HCC. (A, B) Six ferritinophagy‐related genes with differential expression were screened out from The Cancer Genome Atlas (TCGA). (C) Cox proportional hazards model revealed that *FTH1* and *FTL* were significant risk factors, with *FTH1* being more influential. (D) *FTH1* was significantly upregulated in HCC tissues. (E) *FTH1* expression was correlated with patient prognosis. (F‐G) Distribution of patient survival status, *FTH1* heat map, and RTK scores in TCGA. *FTH1* mRNA (H) and protein (I) were highly expressed in HCC tissues. (J) FTH1 protein was highly expressed in HCC cell lines. (K) High and low Ki67 index values for FTH1. (L) Patients with high FTH1 expression have poor prognosis. ****p < 0.0001.

We identified 21 ferritinophagy‐related genes from GeneCards. Six (*TRIM27*, *ALOX15*, *FTL*, *BCAT2*, *FTH1*, and *ZFP36*) were differentially expressed in 374 HCC cases versus 50 normal liver samples from TCGA (Figure 1A,B). The Cox proportional hazards model identified *FTH1* and *FTL* as significant factors, with a more pronounced effect for *FTH1* (Figure 1C). *FTH1* expression was markedly elevated in HCC tissues (Figure 1D). Furthermore, *FTH1* expression negatively correlated with prognosis; patients with *FTH1* upregulation had poorer outcomes (Figure 1E–G). Bioinformatics analyses indicated that *FTH1* expression correlated with B cell activation, CD4+/CD8+ T cell activity, dendritic cells, macrophages, and neutrophil activity (Figure S1).

Analysis of 30 fresh HCC and adjacent non‐cancerous tissues revealed significant *FTH1* mRNA upregulation in the tumour samples (Figure 1H). Western blotting of eight paired HCC and adjacent tissues corroborated the mRNA results (Figure 1I). The upregulation of FTH1 was consistent across the HCC cell lines (Figure 1J). Additional IHC confirmed that the Ki67 index was elevated in tumours with high FTH1 scores (Figure 1K). In line with TCGA results, patients with HCC and high FTH1 expression had worse prognoses (Figure 1L).

### 3.2 *FTH1* Promotes Tumour Progression in HCC Cells

**FIGURE 2:**
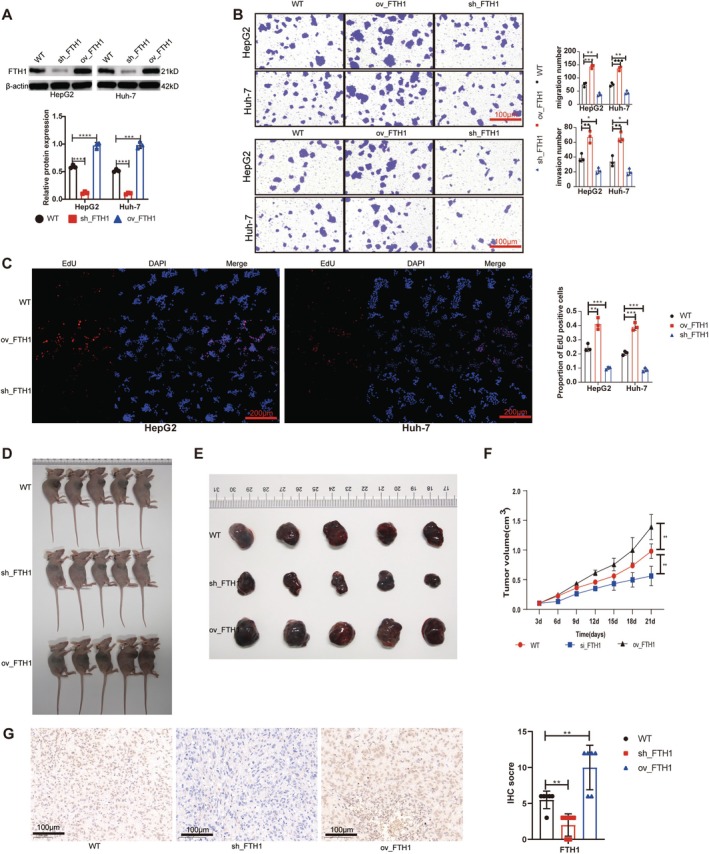
*FTH1* promotes tumour progression in HCC cells. (A) Establishment of *FTH1*‐knockdown and ‐overexpressing HCC cell lines. (B) Migration and invasion ability of HCC cell lines decreased after *FTH1* knockdown and increased after overexpression. (C) Proliferation ability of HCC cell lines decreased after *FTH1* knockdown and increased after overexpression. (D‐F) *FTH1* knockdown decreased tumourigenicity in nude mice subcutaneously treated with HCC cells, and overexpression increased tumour development. (G) Ki67 expression decreased in tumours from HCC mouse model after *FTH1* knockdown, then increased after *FTH1* overexpression. **p < 0.01, ***p < 0.001, ****p < 0.0001.

We successfully established HCC cell lines using *FTH1* knockdown (sh_*FTH1*) and overexpression (ov_*FTH1*) (Figure 2A). *FTH1* knockdown decreased HCC cell proliferation, migration, and invasion capabilities, whereas *FTH1* overexpression enhanced these capabilities (Figure 2B,C). In vivo experiments with nude mice showed that *FTH1* knockdown inhibited tumour growth, while *FTH1* overexpression promoted it (Figure 2D–F). Additionally, the Ki67 index decreased in *FTH1*‐knockdown tumours and increased in *FTH1*‐overexpressing tumours (Figure 2G).

### 3.3 *FTH1* Affects 6‐HMT in the Tryptophan Metabolic Pathway

**FIGURE 3:**
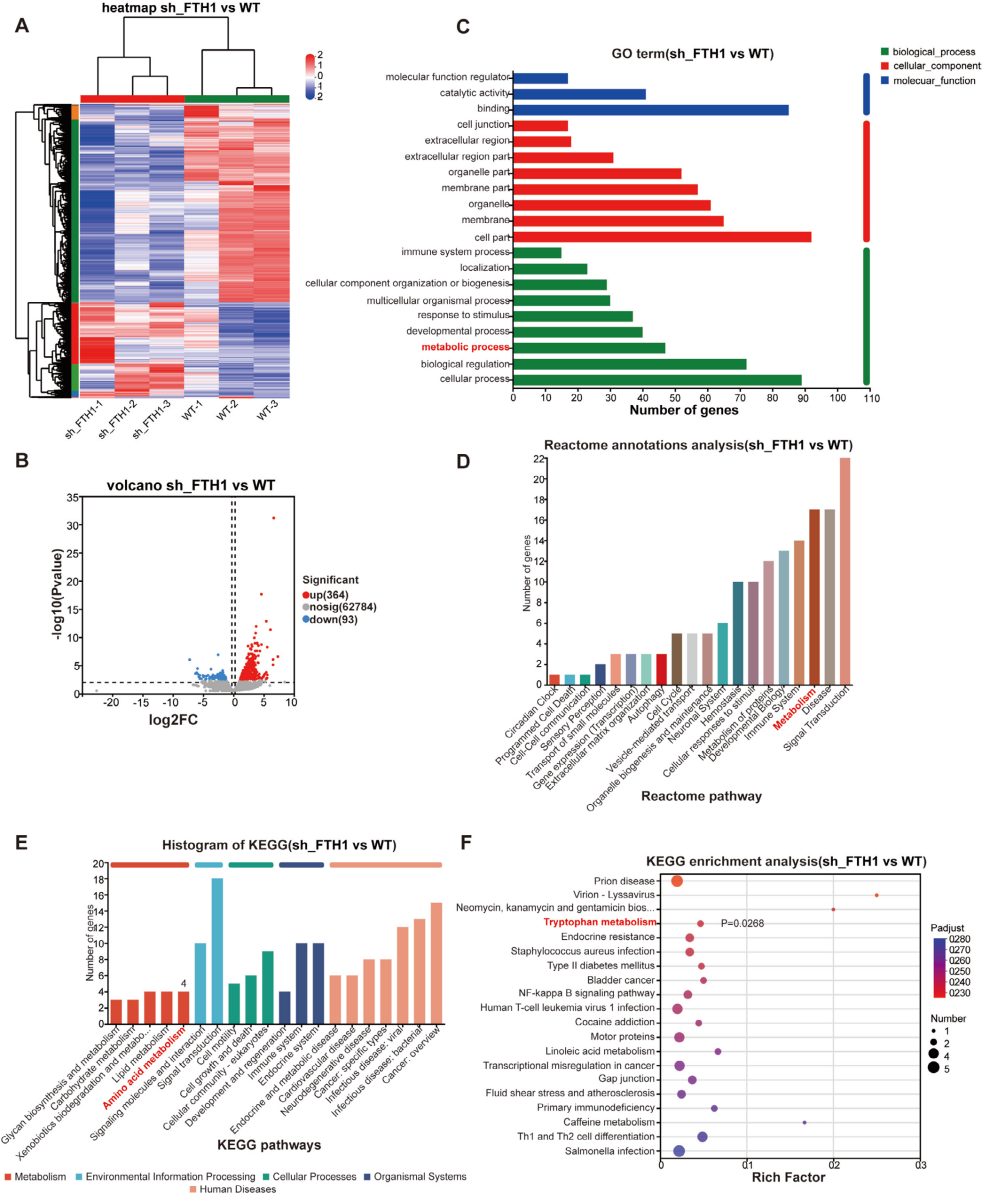
Transcriptome sequencing analysis reveals that *FTH1* is closely related to amino acid metabolism. (A, B) Heatmaps and volcano plots showing differentially expressed genes obtained from transcriptome sequencing of sh_*FTH1* (knockdown) versus WT cell lines. Differentially expressed genes were subjected to GO enrichment analysis (C), Reactome pathway enrichment analysis (D), and KEGG enrichment analysis (E, F).

Transcriptomic sequencing of WT and sh_*FTH1* HepG2 cell lines (three vs. three) revealed 457 differentially expressed genes in the latter group; 93 were upregulated and 364 downregulated from WT levels (Figure 3A,B). Gene Ontology (GO), KEGG, and Reactome analyses revealed that *FTH1* was closely associated with metabolic processes, particularly amino acid metabolism (Figure 3C–E). In particular, KEGG enrichment analysis found that tryptophan metabolism was highly correlated with *FTH1* (Figure 3F). Transcriptome sequencing data revealed that *CYP1A1* and *CYP1A2* were predominantly enriched in the tryptophan metabolic pathway. These enzymes primarily catalyse the conversion of melatonin to 6‐HMT, suggesting that *FTH1* influences 6‐HMT synthesis (Figure S2).

**FIGURE 4:**
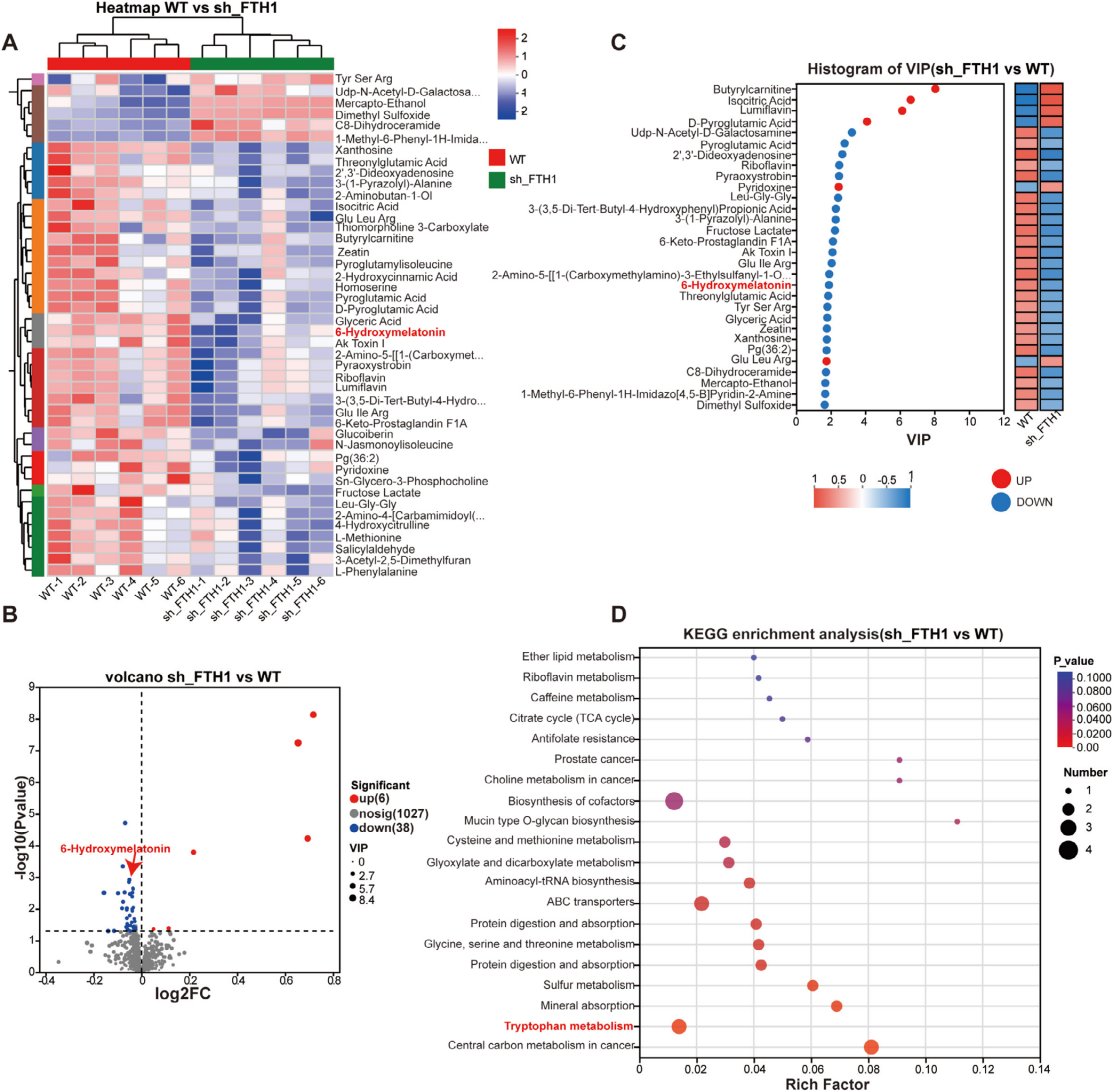
***FTH1*** affects synthesis of tryptophan metabolite 6‐hydroxymelatonin (6‐HMT). (A, B) Heatmaps and volcano plots showing differential metabolites from metabolome sequencing of sh_FTH versus WT cell lines. (C) Histogram of VIP showing a significant difference in 6‐HMT synthesis. (D) The tryptophan metabolic pathway was significantly enriched, according to KEGG pathway analysis.

Metabolomic sequencing indicated that 38 metabolites decreased and six metabolites increased in the sh_*FTH1* group compared with WT (*n* = 6 per group; Figure 4A,B). Notably, and in support of the transcriptome data, *FTH1* knockdown significantly lowered 6‐HMT levels (Figure 4A,C). Furthermore, KEGG pathway analysis demonstrated significant enrichment of the tryptophan metabolism pathway (Figure 4D).

### 3.4 *FTH1* Affects ROS and Cellular Ferroptosis via 6‐HMT

**FIGURE 5:**
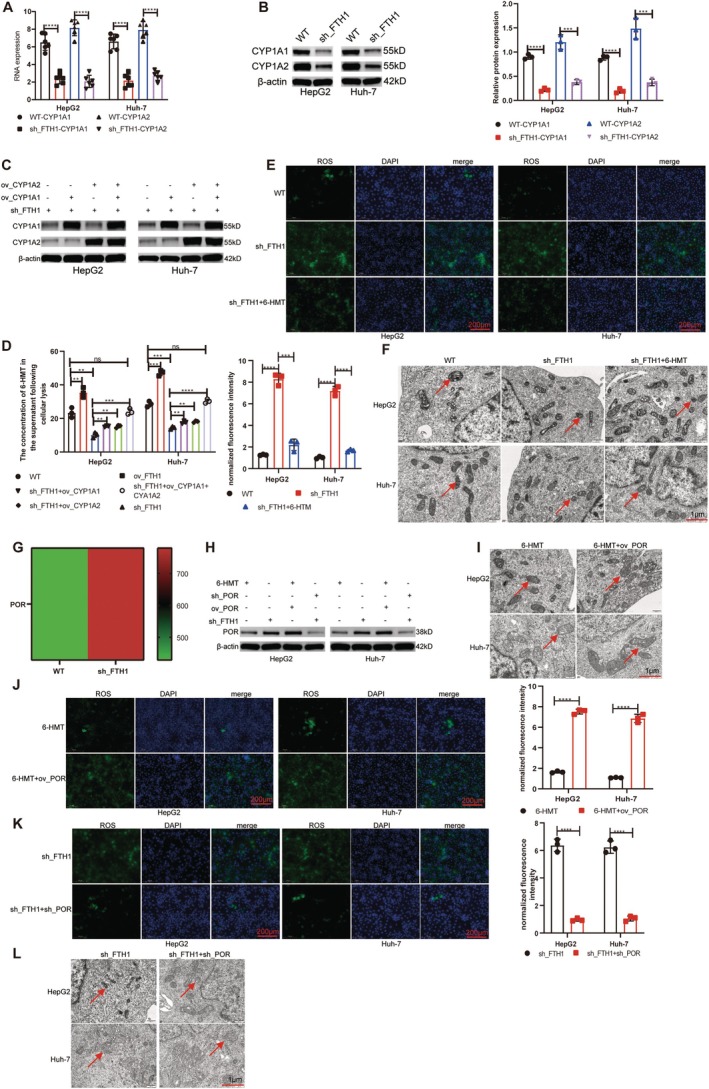
*FTH1* mechanism of action in HCC cell lines. *FTH1* knockdown significantly decreased *CYP1A1* and *CYP1A2* mRNA expression (A) and protein concentrations (B). (C) Cell lines used for establishing the in vitro HCC model. (D) *FTH1* affected 6‐hydroxymelatonin (6‐HMT) synthesis through *CYP1A1* and *CYP1A2*, and both *CYP1A1* and *CYP1A2* were critical. (E) 6‐HMT inhibited the ROS level of HCC cell lines. (F) 6‐HMT suppressed ferroptosis levels in HCC cell lines. (G) Transcriptome sequencing results showed that P450 oxidoreductase (POR) expression levels were significantly increased in cell lines knocking down *FTH1*. (H) 6‐HMT inhibited POR protein expression in cells. (I, J) POR overexpression in cells reversed 6‐HMT‐induced decreases in ROS and ferroptosis. (K, L) POR knockdown restored lower ROS and ferroptosis levels under *FTH1*. **p < 0.01; ***p < 0.001, ****p < 0.0001; ns, not significant.

Based on these findings, we further investigated whether *FTH1* influences *CYP1A1* and *CYP1A2* in HCC cell lines. We found that *FTH1* knockdown significantly downregulated *CYP1A1* and *CYP1A2* mRNA and protein expression (Figure 5A,B). Through this downregulation, *FTH1* knockdown decreased 6‐HMT levels (Figure 5C,D). Moreover, concurrently reintroducing *CYP1A1* and *CYP1A2* following *FTH1* knockdown restored 6‐HMT levels to baseline (Figure 5C,D). Because 6‐HMT inhibited cellular ROS production and ferroptosis, supplementation with 6‐HMT reversed the *FTH1*‐knockdown‐induced increases in ROS levels and ferroptosis (Figure 5E,F).

Transcriptomic sequencing revealed significant alteration in the level of cytochrome P450 oxidoreductase (POR), an enzyme closely associated with ROS production (Figure 5G). Hence, we assessed POR expression after adding 6‐HMT to sh_*FTH1* HCC cell lines and found that 6‐HMT suppressed POR (Figure 5H). POR overexpression in 6‐HMT‐treated HCC cells increased ROS levels and ferroptosis (Figure 5I,J). Following POR knockdown in the sh_*FTH1* HCC cell line, ROS levels and ferroptosis were both decreased (Figure 5K,L).

### 3.5 Tryptophan Upregulation in HCC Cells Inhibits the NCOA4‐FTH1 Autophagy Pathway

**FIGURE 6:**
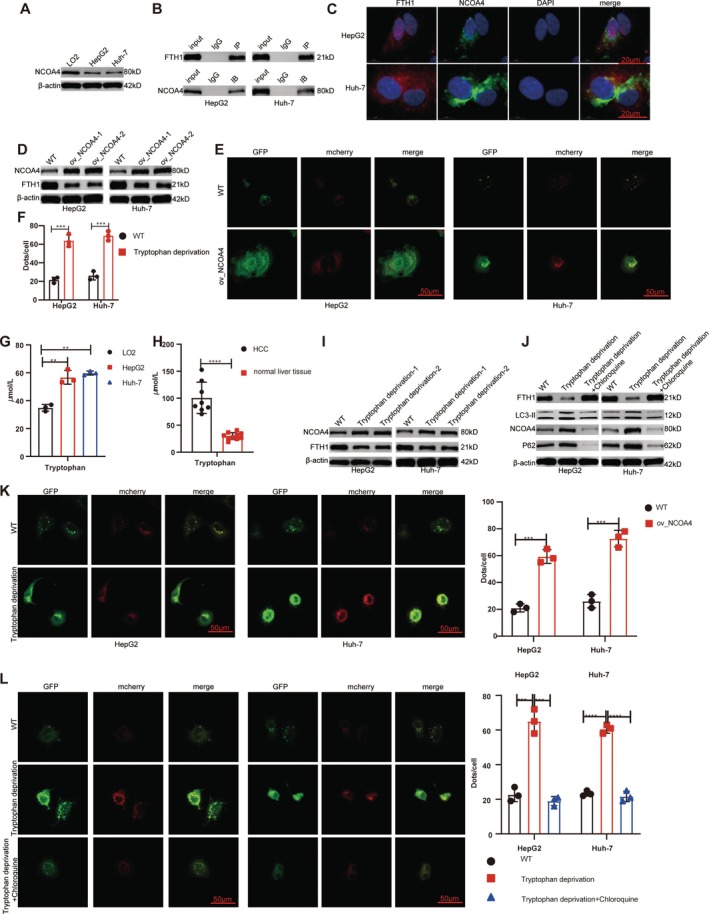
Tryptophan upregulation in HCC cells inhibits the NCOA4‐FTH1 autophagy pathway. (A) NCOA4 concentrations decreased significantly in HCC cell lines. (B) Co‐IP demonstrated a clear interplay between NCOA4 and FTH1 proteins. (C) Immunofluorescence revealed that NCOA4 and FTH1 proteins were co‐expressed in HCC cell lines. (D) FTH1 protein concentration decreased significantly after overexpressing NCOA4 in HCC cell lines. (E, F) Autophagy flow assay demonstrated that cell‐selective autophagy increased significantly with NCOA4 overexpression. Tryptophan levels were significantly elevated in HCC cell lines (G) and HCC tissues (H). (I) Tryptophan deprivation upregulated NCOA4 and downregulated FTH1. (J) Selective autophagy inhibitors reversed the increase in cell‐selective autophagy induced by tryptophan deprivation. (K, L) Autophagy flow experiments verified that tryptophan modulated selective autophagy in HCC cells. ***p < 0.001, ****p < 0.0001.

NCOA4, a selective autophagy adapter protein, was expressed significantly less in HCC cell lines than in normal hepatocytes (Figure 6A). Results from Co‐IP experiments revealed a distinct reciprocal relationship between NCOA4 and FTH1 in HCC (Figure 6B). Immunofluorescence assays confirmed NCOA4 and FTH1 co‐expression in HCC cell lines (Figure 6C), with NCOA4 overexpression significantly decreasing FTH1 levels (Figure 6D). Additionally, autophagy flux assays demonstrated that NCOA4 overexpression markedly increased autophagy (Figure 6E,F).

We next assessed tryptophan levels in HCC cell lines and tissues, demonstrating that they were significantly higher than in normal liver cell lines and tissues (Figure 6G,H). Furthermore, HCC cell lines cultured in tryptophan‐deprived medium exhibited NCOA4 upregulation and FTH1 downregulation (Figure 6I). Upon addition of the protein‐selective autophagy inhibitor chloroquine, we observed a reduction in NCOA4, an increase in FTH1 levels, and a decrease in the autophagy markers LC3‐II and P62 (Figure 6J). Autophagy flow experiments further confirmed the importance of tryptophan as a regulatory metabolite in cell‐selective autophagy (Figure 6K,L).

## 4 Discussion

In this study, we utilised publicly available transcriptomic data from TCGA, along with associated clinical and prognostic information, to clarify the mechanisms of ferritinophagy in HCC. In the Cox proportional hazards model, both *FTH1* and *FTL* emerged as prognostic indicators. However, *FTH1* demonstrated a more statistically significant *p*‐value relative to *FTL*. Moreover, *FTH1* serves as the active component of ferritin, playing an essential role in ferroxidase activity, which directly impacts iron homeostasis and ferroptosis. Studies indicate that *FTH1* is involved in ferritinophagy, a selective autophagic pathway that leads to the degradation of ferritin by ferroptosis [17]. As a subunit of ferritin, FTH1 is a principal component in the storage and release of ferric ions, which is a critical process for physiological function [18]. Ferritinophagy is thought to increase ferroptosis of neurons in neurodegenerative diseases [19]. Changes to *FTH1* expression also appear to influence tumour cell proliferation and metabolism, suggesting that FTH1 is a viable therapeutic target for various diseases [20]. *FTH1* expression has been reported to be upregulated in HCC [21]. The results are similar to what we have observed. Our analysis of TCGA data indicates that *FTH1* upregulation is associated with a worse HCC prognosis. A significant decrease in FTH1 indicates that osteosarcoma cells are more sensitive to chemotherapy [22]. FTH1 is considered a potential target for treatment via ferroptosis and acts as an oncogene in the initiation and progression of HCC [23].

In addition to examining existing data, we applied lentiviral vectors and transfection procedures [24] to develop *FTH1*‐knockdown and ‐overexpressing HCC cell lines. Our findings indicate that *FTH1* knockdown decelerates tumour progression, whereas *FTH1* overexpression enhances tumorigenic capacity, suggesting that *FTH1* facilitates malignant progression in HCC cells.

To elucidate the underlying mechanisms, we conducted transcriptome sequencing of WT and sh_*FTH1* cell lines, followed by functional analysis of differentially expressed genes. The results revealed that *FTH1* is involved in tryptophan metabolism. Specifically, *FTH1* affected the expression of CYP1A1 and CYP1A2, cytochrome P450 (CYP450) family members that play major roles in drug metabolism, carcinogen activation, and transformation of endogenous compounds [25], including tryptophan [26]. CYP1A1 and CYP1A2 are monooxygenases with an N‐terminal signal peptide, a heme‐binding domain, and a C‐terminal vascular endothelial growth factor‐binding domain [27]. In the tryptophan metabolic pathway, CYP1A1 and CYP1A2 act jointly to catalyse the conversion of the tryptophan‐derivative melatonin into 6‐HMT. We hypothesised that *FTH1* modulates 6‐HMT synthesis via its effects on *CYP1A1* and *CYP1A2* in HCC cells. Knockdown experiments using HCC cell lines validated our hypothesis: *FTH1* knockdown inhibited 6‐HMT synthesis by downregulation of *CYP1A1* and *CYP1A2*. Concurrent overexpression of *CYP1A1* and *CYP1A2* restored 6‐HMT synthesis following *FTH1* knockdown.

Notably, 6‐HMT functions as a potent agonist of melatonin receptors, including MT1 and MT2 [28]. This binding effect may modulate melatonin‐related physiological functions, including circadian rhythms, sleep–wake cycles, and the immune system [29]. Melatonin exhibits antioxidant properties, scavenging free radicals and terminating free radical chain reactions. Given its structural similarity to melatonin, 6‐HMT may also possess antioxidant activity [30, 31] and potentially play a role in ferroptosis. We tested this hypothesis in HCC cells and demonstrated that 6‐HMT inhibited ROS production and ferroptosis. The exact mechanisms of this process require further investigation, although our transcriptomic analyses strongly indicate that POR is involved.

Specifically, POR expression was upregulated following sh_*FTH1* treatment. Located in the endoplasmic reticulum membrane [16], POR is crucial to metabolic reactions of hormones, drugs, and exogenous substances catalysed by cytochrome P450 proteins [32]. POR is a flavoprotein characterised by a domain that binds to the cofactor reduced nicotinamide adenine dinucleotide (NADH) and a domain analogous to flavodoxin that binds to the cofactor flavin adenine dinucleotide (FAD). This structural configuration enables direct electron transfer from NADPH to microsomal P450 enzymes [33]. *POR* mutations cause a complex spectrum of disorders, including defects in P450C17 and P450C21, amenorrhea, disordered steroid hormone production, congenital adrenal hyperplasia, and Aintree–Bixler syndrome. These diseases resemble conditions caused by abnormalities in steroid‐metabolising enzymes such as aromatase, 21‐hydroxylase, and 17α‐hydroxylase [34]. Additionally, POR redox reactions generate ROS as byproducts, thereby inducing lipid peroxidation and ferroptosis [35]. In summary, our findings indicate that 6‐HMT suppresses *POR* expression. Additionally, *POR* overexpression reverses 6‐HMT‐induced decreases in ROS and ferroptosis. This is a novel mechanism of action, whereby *FTH1* influences cellular ROS and ferroptosis via an amino acid metabolic pathway.

Given the pivotal role of tryptophan metabolism in *FTH1* function, we analysed tryptophan levels in HCC cell lines and tissues. Tryptophan levels were significantly higher in HCC cell lines and tissues than in normal hepatocytes and liver tissue. Tryptophan metabolism primarily involves the kynurenine, 5‐hydroxytryptamine, and indole pathways [36]. Tryptophan derivatives play crucial roles in regulating physiological functions such as inflammation, metabolism, immune responses, and neurological function [37]. Not surprisingly, an expanding body of evidence indicates a robust association between disordered tryptophan metabolism and disease [16]. Indeed, regulation of tryptophan metabolism can modulate disease progression in many cases. For instance, liver tumours require substantial amounts of tryptophan to synthesise the oncogenic metabolite indole‐3‐pyruvate, and *MYC* induction is pivotal to tryptophan involvement in HCC tumour metabolism [38]. Here, we investigated the effect of tryptophan on the NCOA4‐FTH1 pathway and found that tryptophan was an inhibitor, suppressing selective FTH1 autophagy (leading to FTH1 upregulation), similar to the outcome when cells were treated with the autophagy inhibitor chloroquine. Tryptophan deprivation reversed this inhibition of FTH1 autophagy and thus decreased FTH1 levels.

## 5 Conclusion

We identified FTH1 as a ferritinophagy‐associated protein important to HCC progression. *FTH1* upregulation was linked to poor patient prognoses. Our findings indicate that targeting selective FTH1 autophagy may be a promising direction for developing new therapies. To that end, we demonstrated that the NCOA4 pathway is involved in FTH1 autophagy and is modulated by intracellular tryptophan levels. Moreover, FTH1 autophagy affects the synthesis of the tryptophan metabolite 6‐HMT, which regulates cellular ROS and ferroptosis levels. This result provides a hint of how *FTH1* influences HCC pathogenesis and positions the tryptophan metabolism pathway as a promising therapeutic target in HCC.

## Study Limitations

While our findings advance the understanding of the role of *FTH1* in HCC, several limitations need to be pointed out. First, in vitro and xenograft models do not fully recapitulate the complexity of the human tumour microenvironment, including immune interactions. Secondly, while we identified CYP1A1/2 as downstream effectors of *FTH1*, the precise molecular mechanisms linking *FTH1* to these enzymes remain unclear. Finally, the study focused on NCOA4‐mediated autophagy, leaving other potential regulatory pathways unexplored.

## Future Directions

To address these limitations, future studies for mechanistic investigations using CRISPR screens or co‐immunoprecipitation coupled with mass spectrometry could elucidate how FTH1 interacts with CYP1A1/2 and other partners. Advanced models, such as organoids or immunocompetent murine systems, would better mimic in vivo conditions. Exploring dietary or systemic tryptophan modulation in HCC progression could clarify its therapeutic relevance. Finally, developing small‐molecule inhibitors targeting FTH1‐NCOA4 interactions or 6‐HMT synthesis may pave the way for preclinical trials. These efforts will deepen the understanding of ferritinophagy in HCC and expand the arsenal of targeted therapies for this lethal disease.

## Author Contributions

All authors contributed to the study's conception and design. Y.Y., S.H., Y.L., and X.C. performed the experimental operation and collected the data. X.G., C.Z., and X.Y. designed the experiment and analysed the data. Q.N., Z.Y., M.Z., and W.C. wrote the first draft of the manuscript and further edited upon input from all co‐authors, and all authors commented on previous versions. All authors read and approved the final manuscript.

## Disclosure

The authors have nothing to report.

## Conflicts of Interest

The authors declare no conflicts of interest.

## Supporting information


**Data S1.** Supporting Information.

## Data Availability

The data that support the findings of this study are available from the corresponding author upon reasonable request.
