# Supplementary material for: Tryptophan Suppresses FTH1 ‐Driven Ferritinophagy, a Key Correlate of Prognosis in Hepatocellular Carcinoma
Source: Cell Prolif. 2025 Jun 12;59(1):e70074. doi: 10.1111/cpr.70074 (PMC12774622; doi:10.1111/cpr.70074)
Supplement: Supplementary file 1 — Data S1. Supporting Information. [file CPR-59-e70074-s001.docx]

**Supporting information**

**Figure S1. Gene-immune cell interactions**


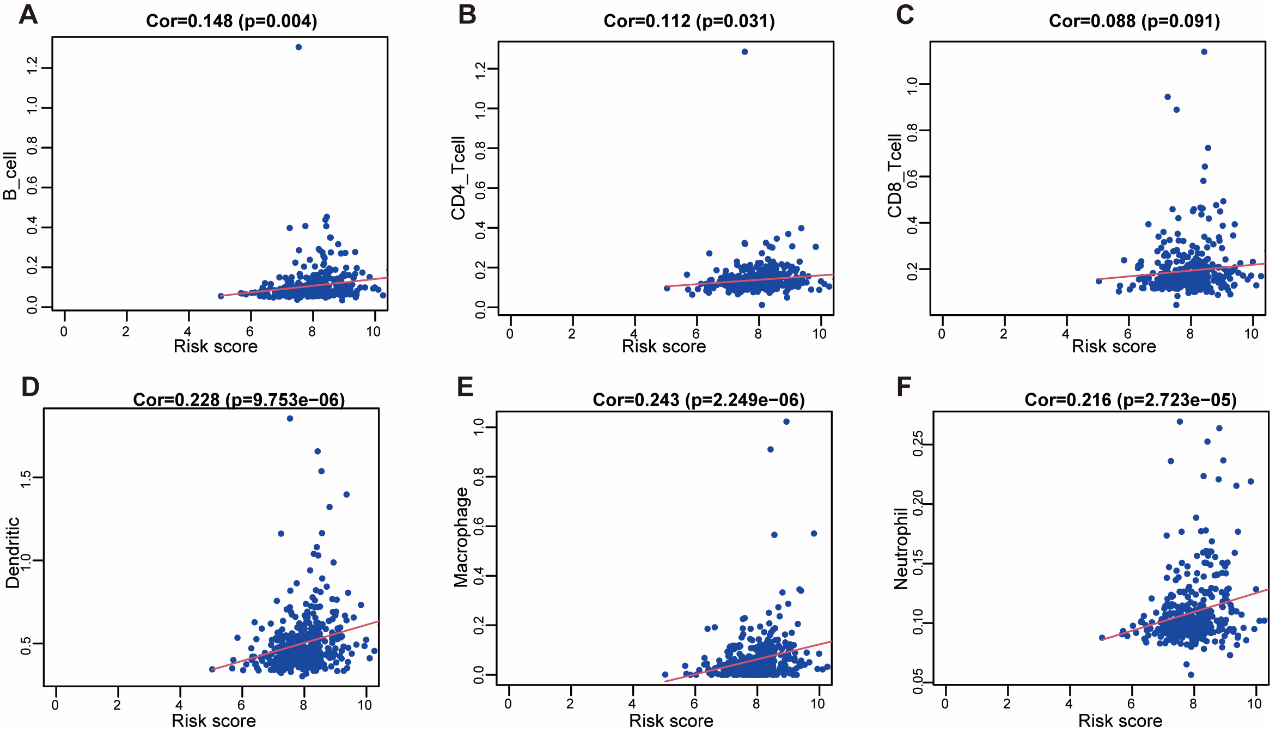


*FTH1* expression correlated with B-cell activity (A), CD4+ T-cell activity (B), CD8+ T-cell activity (C), dendritic cell activity (D), macrophage activity correlation (E), and neutrophil activity (F).

**Figure S2. *FTH1* acts on *CYP1A1* and *CYP1A2* to influence *6-hydroxymelatonin* synthesis.**


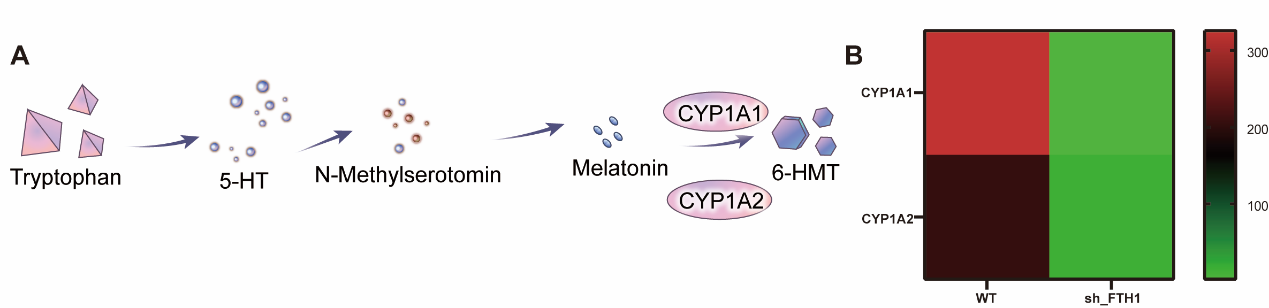


(A) Schematic representation of tryptophan conversion to 6-hydroxymelatonin. (B) Schematic representation of *CYP1A1* and *CYP1A2* mRNA expression from transcriptome sequencing.

Supplementary Table 1．Primer sequences and amplified fragment lengths in PCR amplification reactions

| gene | primer sequence | length |
| --- | --- | --- |
| *FTH1* | 5'-CCCCCATTTGTGTGACTTCAT-3' | 180bp |
|  | 5'-GCCCGAGGCTTAGCTTTCATT-3' |  |
| *CYP1A1* | 5'-TCGGCCACGGAGTTTCTTC-3' | 141bp |
|  | 5'-GGTCAGCATGTGCCCAATCA-3' |  |
| *CYP1A2* | 5'-CTGGGCACTTCGACCCTTAC-3' | 99bp |
|  | 5'-TCTCATCGCTACTCTCAGGGA-3' |  |
| *GAPDH* | 5'-ATCATCCCTGCCTCTACTGG-3 | 122bp |
|  | 5'-GTGTCAGTGGTGGACCTGA-3 |  |
